# Depression and quality of life among Macau residents in the 2022 COVID-19 pandemic wave from the perspective of network analysis

**DOI:** 10.3389/fpsyg.2023.1164232

**Published:** 2023-04-24

**Authors:** Tong Leong Si, Pan Chen, Ling Zhang, Sha Sha, Mei Ieng Lam, Ka-In Lok, Ines Hang Iao Chow, Jia-Xin Li, Yue-Ying Wang, Zhaohui Su, Teris Cheung, Gabor S. Ungvari, Chee H. Ng, Yuan Feng, Yu-Tao Xiang

**Affiliations:** ^1^Unit of Psychiatry, Department of Public Health and Medicinal Administration, and Institute of Translational Medicine, Faculty of Health Sciences, University of Macau, Macao, Macao SAR, China; ^2^Centre for Cognitive and Brain Sciences, University of Macau, Macao, Macao SAR, China; ^3^The National Clinical Research Center for Mental Disorders and Beijing Key Laboratory of Mental Disorders, Beijing Anding Hospital and The Advanced Innovation Center for Human Brain Protection, Capital Medical University, Beijing, China; ^4^Kiang Wu Nursing College of Macao, Macau, Macao SAR, China; ^5^Faculty of Health Sciences and Sports, Macao Polytechnic University, Macao, Macao SAR, China; ^6^School of Public Health, Southeast University, Nanjing, China; ^7^School of Nursing, Hong Kong Polytechnic University, Hong Kong, Hong Kong SAR, China; ^8^University of Notre Dame Australia, Fremantle, WA, Australia; ^9^Division of Psychiatry, School of Medicine, University of Western Australia/Graylands Hospital, Mount Claremont, WA, Australia; ^10^Department of Psychiatry, The Melbourne Clinic and St Vincent's Hospital, University of Melbourne, Richmond, VIC, Australia

**Keywords:** depression, quality of life, prevalence, COVID-19, network analysis

## Abstract

**Background:**

In the summer of 2022, Macau experienced a surge of COVID-19 infections (the 618 COVID-19 wave), which had serious effects on mental health and quality of life (QoL). However, there is scant research on mental health problems and QoL among Macau residents during the 618 COVID-19 wave. This study examined the network structure of depressive symptoms (hereafter depression), and the interconnection between different depressive symptoms and QoL among Macau residents during this period.

**Method:**

A cross-sectional study was conducted between 26th July and 9th September 2022. Depressive symptoms were measured with the 9-item Patient Health Questionnaire (PHQ-9), while the global QoL was measured with the two items of the World Health Organization Quality of Life-brief version (WHOQOL-BREF). Correlates of depression were explored using univariate and multivariate analyses. The association between depression and QoL was investigated using analysis of covariance (ANCOVA). Network analysis was used to evaluate the structure of depression. The centrality index “Expected Influence” (EI) was used to identify the most central symptoms and the flow function was used to identify depressive symptoms that had a direct bearing on QoL.

**Results:**

A total 1,008 participants were included in this study. The overall prevalence of depression was 62.5% (n = 630; 95% CI = 60.00–65.00%). Having depression was significantly associated with younger age (OR = 0.970; *p* < 0.001), anxiety (OR = 1.515; *p* < 0.001), fatigue (OR = 1.338; *p* < 0.001), and economic loss (OR = 1.933; *p* = 0.026). Participants with depression had lower QoL F (1, 1,008) =5.538, *p* = 0.019). The most central symptoms included PHQ2 (“Sad Mood”) (EI: 1.044), PHQ4 (“Fatigue”) (EI: 1.016), and PHQ6 (“Guilt”) (EI: 0.975) in the depression network model, while PHQ4 (“Fatigue”), PHQ9 (“Suicide”), and PHQ6 (“Guilt”) had strong negative associations with QoL.

**Conclusion:**

Depression was common among Macao residents during the 618 COVID-19 wave. Given the negative impact of depression on QoL, interventions targeting central symptoms identified in the network model (e.g., cognitive behavioral therapy) should be developed and implemented for Macau residents with depression.

## 1. Introduction

Since COVID-19 was first reported in China in early 2020, approximately 600 million people had been infected globally as of the end of 2022 (World Health Organization, 2022). Located on the west of the Pearl River Delta estuary in southern China, Macau is a Special Administrative Region of China (Macau SAR) with a different political and economic system from mainland China. However, Macao adopted a similar COVID management strategy as mainland China (Chen et al., 2023), since the local economy relies heavily on tourism and gambling revenues from mainland China (McCartney, 2021). The latest surge of the COVID-19 infections occurred from June 18 to August 7, 2022 in Macau, known as the “618 COVID-19 wave” (Portal do Governo da Região Administrativa Especial de Macau da República Popular da China, 2022), which caused 1,816 confirmed cases and 6 deaths (Exmoo News, 2022; Macao Daily, 2022d). To prevent the spread of the virus, the quarantine-free border policy between Zhuhai and Macau was suspended, while in Macau the territory lockdown measures were implemented (Radio Television Hong Kong, 2022; Macao Daily, 2022e). From July 11 to July 23, 2022 Macau adopted a “relative standstill” policy; i.e., all businesses and industry activities (including casinos and construction projects) were suspended (Macao Daily, 2022a,b,c). Following the COVID strategy in mainland China, Macau government implemented a Dynamic Zero-COVID policy (Zhao et al., 2022a) and adopted stringent health management and quarantine measures when necessary, which might increase the risk of mental health problems (Zhong et al., 2020; Bai et al., 2022a) such as depressive symptoms (depression hereafter) (Xiong et al., 2020).

Depression is one of the most common mental health problems, especially during the COVID-19 pandemic. Previous studies found that the prevalence of depression were 38.5% (95% CI: 35.5%–41.5%) among Macau residents (Bai et al., 2022b) and 35.2% (95% CI, 32.2–38.3%) among Chinese college students (Li et al., 2020) in the early stage of the pandemic. In contrast, several studies before the COVID-19 pandemic on the epidemiology of depression in Macau found that the prevalence rate was 8.0% in the general population (Hall et al., 2017), 8.6% in older men (Chan and Zeng, 2011) and 11.9% in older women (Chan and Zeng, 2009). Depression is associated with a number of negative outcomes such as impaired functioning (Geiselman and Bauer, 2000; Wieman et al., 2022), lowered quality of life (QoL) (Bertha and Balázs, 2013), and an increased risk of suicidality (Hegerl, 2016; Chen et al., 2022). In order to facilitate health resource allocation and develop appropriate preventive strategies to reduce the likelihood of depression and its negative outcomes, it is important to understand the epidemiology and associated factors of depression.

Most research on depression was traditionally based on the common cause framework (Cramer et al., 2010; Schmittmann et al., 2013; Fried, 2015), in which all symptoms originate from the underlying disease (Lux and Kendler, 2010; Fried, 2015) and all symptoms are clinically equivalent and interchangeable (Cramer et al., 2010; Lux and Kendler, 2010). However, the complexities and ongoing interaction between different depressive symptoms could not be examined from such perspective (Marchetti, 2019; Mullarkey et al., 2019).

In recent years, the network approach has been a novel approach that could conceptualize psychological phenomena (Borsboom and Cramer, 2013). According to network theory, depressive states may result from the co-occurrence of depressive symptoms and the interactions without latent factors (Borsboom, 2008; Borsboom and Cramer, 2013; Santos et al., 2017). Individual psychiatric symptoms are viewed as nodes, while the associations between nodes are viewed as edges in a network model (Epskamp et al., 2012). Network analysis could help determine nodes that are the most central (influential) in the network model and could be targeted for prevention and intervention (Borsboom and Cramer, 2013; Cramer et al., 2016; Marchetti, 2019). In the past years network analysis has been widely applied in different populations during the COVID-19 pandemic such as adolescents (Cai et al., 2022a), the general population (Cheung et al., 2021; Zavlis et al., 2021), older people (Jin et al., 2022), college students (Bai et al., 2021), clinicians (Cai et al., 2022c), and psychiatric patients (Kim et al., 2022). Evidence showed that the pattern and clinical features of depression were highly dependent on the socioeconomic context (Kleinman, 2004; Compton et al., 2006); therefore, the network structure of the depressive symptoms should be examined separately for the population living in areas with different socioeconomic contexts (Cheung et al., 2021). However, no network analysis of depression in Macau residents during the 618 COVID-19 wave has been published.

This study investigated the prevalence and the related factors of depression among Macau residents during the 618 COVID-19 wave, examined the central symptoms of the depression network model, and explored the association between depressive symptoms and QoL. We hypothesized that depression would be common and negatively associated with QoL among Macau residents in this wave.

## 2. Methods

2.

### 2.1. Participants and procedure

This was a cross-sectional study conducted between 26^th^ July 2022 and 9^th^ September 2022 using snowball sampling method. A Quick Response code (QR code) linked with the invitation and the study assessment was distributed *via* major social media platforms including WeChat, Facebook and Instagram in Macau. To be eligible, participants met the following inclusion criteria: (1) aged 18 years or above, and able to understand the purpose and content of the assessments; (2) Macau residents living in Macau during the 618 COVID-19 wave. There were no exclusion criteria in this study. Due to the risk of COVID-19 infection, face-to-face assessments were not adopted. Following other studies (Bai et al., 2022b; Cai et al., 2022b) the “Questionnaire Star” program was conducted in data collection. The participants provided online written informed consent on a voluntary and confidential basis. The study protocol was approved by the Institutional Review Board (IRB) of the University of Macau.

### 2.2. Measures

Socio-demographic information, such as age, gender, marital status, education level, employment status, and monthly income during the COVID-19 wave, was collected. Variables related to COVID-19 were also measured, including the level of economic loss caused by the COVID-19 wave, concerns about the COVID-19 pandemic, being quarantined, and having COVID-19 infection, fatigue, and regular physical exercise during the wave. In addition, the presence of chronic physical diseases, history of psychiatric disorders, and suicidality during the COVID-19 wave were also collected.

The validated Chinese version of the 9-item Patient Health Questionnaire (PHQ-9) was used to measure the presence and severity of depressive symptoms (Kroenke et al., 2001; Chen et al., 2015), which consisted of nine dimensions: including (1) Anhedonia; (2) Sad Mood; (3) Sleep; (4) Fatigue; (5) Appetite; (6) Guilt; (7) Concentration; (8) Motor disturbances; and (9) Suicide. The PHQ-9 items are developed based on the Major depressive disorder criteria from the Diagnostic and Statistical Manual of Mental Disorders-IV (DSM-IV). Each item ranges from 0 (not at all) to 3 (nearly every day), with the total score ranging between 0 and 27. Following the previous study of Wang et al. (2014), a PHQ-9 total score of ≥5 was considered “having depressive symptoms”; specifically, a total score of 5, 10, 15, and 20 were used as the cut-off values for having mild depressive symptoms”, “moderate depressive symptoms”, “moderately severe depressive symptoms” and “severe depressive symptoms respectively (Kroenke et al., 2001). The psychometric properties of PHQ-9 Chinese version in Chinese populations are considered satisfactory.

The severity of anxiety was assessed using the validated Chinese version of the seven-item Generalized Anxiety Disorder scale (GAD-7) (Spitzer et al., 2006; He et al., 2010). The GAD-7 comprises 7 items with each scored from 0 (not at all) to 3 (nearly every day), and the total score ranges from 0 to 21 with higher scores indicating more severe anxiety symptoms. Global quality of life (QoL) was measured with the total score of the first two items of the World Health Organization Quality of Life-brief version (WHOQOL-BREF) (The WHOQOL Group, 1998; Skevington et al., 2004), with higher total scores indicating higher QoL. The Chinese version of the WHOQOL-BREF has been validated in Chinese populations (Hao et al., 2006; Xia et al., 2012).

### 2.3. Statistical analysis

#### 2.3.1. Univariate and multivariate analyses

SPSS version 26.0 (SPSS Inc., Chicago, Illinois, USA) was used to conduct univariate and multivariate analyses. Continuous variables were tested for normal distributions using Kolmogorov–Smirnov tests and Q-Q plots. Socio-demographic and clinical data, and COVID-19-related variables between “with depression” and “without depression” groups were analyzed using Chi-square test, Student’s t-test or Mann–Whitney U tests, as appropriate. Analysis of covariance (ANCOVA) was used to compare QoL between “with depression” and “without depression” groups, after controlling for variables with significant differences in univariate analysis. Binary logistic regression analysis with the “Enter” method was used to test independent correlates of depression. Those with significant group differences in univariate analyses were entered as independent variables, while having depression was the dependent variable. The significance level was set at *p* < 0.05 for all tests (two-tailed).

#### 2.3.2. Network structure

The R software (R Core Team, 2013) was used to conduct the network analysis. In the network model, each symptom is represented as a node, and the association between two nodes is represented as an edge (Beard et al., 2016). The estimate and visualization of the network were performed using the R-packages “qgraph” (Version 1.6.5) (Epskamp et al., 2012) and “bootnet” (Version 1.4.3) (Epskamp et al., 2018). Edges in the network were shrunk and relevant tuning parameters were selected using the least absolute shrinkage and selection operator (LASSO) and extended Bayesian information criteria (EBIC) to make the symptom network sparser and simpler to understand (Epskamp et al., 2018), which could improve the accuracy of the prediction, sparse, and interpretability of the network model (Yang et al., 2022). The correlation between two nodes in green color indicated positive correlations, while red color indicated negative correlations, with thicker edges representing stronger correlations. To determine central (influential) symptoms in the network, expected Influence (EI) as a reliable centrality index was adopted (Robinaugh et al., 2016). The predictability of each node was estimated using the R package “mgm” (Haslbeck and Waldorp, 2015), which is defined as the variance of a node explained by all other nodes in the network model. Further, the “flow” function in R package “qgraph” was conducted to identify individual depressive symptoms that were directly associated with QoL (Epskamp et al., 2012).

To evaluate the robustness of the results, the stability and accuracy of the network were evaluated using the R package “bootnet” (version 1.4.3) (Epskamp et al., 2018). Case-dropping bootstrap was used to estimate the stability of the network. The network was deemed stable if samples could be removed from the dataset without causing significant changes in the node’s centrality index. Stability was graphically represented and quantified by calculating the Correlation Stability Coefficient (CS-C), with moderate stability indicated by values higher than 0.25, while strong stability indicated by values larger than 0.5 (Koo and Li, 2016). Bootstrapped 95% confidence intervals (Cis) were used to measure edge accuracy, with a narrower CI indicating a more reliable network (Epskamp et al., 2018).

## 3. Results

### 3.1. Participant characteristics

Of a total of 1,020 Macau residents invited to participate in this study, 1,008 (739 female, 269 male) met the study entry criteria and were included in this study, giving a participation rate of 98.8%. The mean age of participants were 34.85 (SD = 11.5) years, while 831 (82.4%) had high education (i.e., undergraduate/college or above), 687 (68.2%) were employed during the COVID-19 wave, 471 (46.7%) were married, and 935 (92.8%) lived with others.

### 3.2. Prevalence and correlates

The overall prevalence of depression (PHQ-9 total score ≥ 5) was 62.5% (n = 630; 95% CI = 60.00–65.00%); specifically, 343 (34.0%; 95% CI = 31.10–36.96%) had mild depression, while 163 (16.2%; 95% CI = 13.89–18.45%) had moderate depression 74 (7.3%; 95% CI = 5.73–8.95%) had moderately severe depression, and 50 (5.0%; 95% CI = 3.62–6.30%) had severe depression. Table 1 summarizes the demographic characteristics of participants with depression and those without depression.

**Table 1:** Comparison between Macau residents with and without depressive symptoms with respect to demographic and clinical variables.

Variables	Without depression (*N* = 630)		With depression (*N* = 378)		Univariable analysis		
	*n*	%	*n*	%		df	*p*
Male	162	25.7	107	28.3	0.812	1	0.368
Married	190	50.3	281	44.6	3.042	1	0.081
Living with others	346	91.5	589	93.5	1.348	1	0.246
College education and above	308	81.5	523	83.0	0.384	1	0.535
Employed during the COVID-19 pandemic	263	69.6	424	67.3	0.563	1	0.453
Very concerned of the COVID-19 pandemic	271	71.7	493	78.3	5.543	1	**0.019**
Being quarantined during the COVID-19 pandemic	36	9.5	73	11.6	1.043	1	0.307
Worried about COVID-19 infection					19.603	2	**<0.001**
No worry	168	44.4	228	36.2			
Worried	177	46.8	285	45.2			
Very worried	33	8.7	117	18.6			
Economic loss					71.446	2	**<0.001**
No or minimal	183	48.4	187	29.7			
Fair	159	42.1	246	39.0			
Very much	36	9.5	197	31.3			
Monthly income (≥ MOP 30,000)	170	45.0	211	33.5	13.246	1	**<0.001**
Physical exercise during the pandemic (≥ 30 min/day)	201	53.2	270	42.9	10.103	1	**0.001**
Presence of chronic physical diseases	10	2.6	24	3.8	0.982	1	0.322
Having a history of psychiatric disorders	5	1.3	53	8.4	21.899	1	**<0.001**
Any suicidality during the latest COVID-19 wave	6	1.6	84	13.3	40.086	1	**<0.001**
	Mean	SD	Mean	SD	*t/Z*	df	*p*
Age (years)	37.17	12.626	33.46	10.565	−4.279	---^a^	**<0.001**
GAD-7 total	1.63	2.274	7.544	5.183	−20.102	---^a^	**<0.001**
Fatigue	3.73	2.251	6.50	2.038	−16.846	---^a^	**<0.001**
Global quality of life	6.83	1.256	5.62	1.405	−13.057	---^a^	**<0.001**

a = Mann–Whitney U test; Bolded values are *p* < 0.05; df: degree of freedom; PHQ-9: Patient Health Questionnaire-9 items; GAD-7: Generalized Anxiety Disorder-7 items; PCL: PTSD Check List; SD: standard deviation. 1 USD = 8.078 MOP.

**Table 2:** Independent correlates of depressive symptoms among Macau residents during the 618 COVID-19 wave (*N* = 1,008).

Variables	Multiple logistic regression analysis		
	*p*	*OR*	95% *CI*
Very concerned of the COVID-19 pandemic	0.519	0.871	0.572–1.326
Worried about COVID-19 infection			
No worry	–	–	–
Worried	0.984	1.004	0.682–1.479
Very worried	0.756	1.103	0.594–2.048
Economic loss			
No or minimal	–	–	–
Fair	0.508	1.140	0.774–1.677
Very much	**0.026**	1.933	1.083–3.451
Monthly income (≥ MOP 30,000)	0.303	0.806	0.536–1.214
Physical exercise during the pandemic (≥ 30 min/day)	0.138	0.762	0.532–1.092
Having a history of psychiatric disorders	0.434	1.665	0.464–5.973
Any suicidality during the latest COVID-19 wave	0.735	1.208	0.404–3.608
Age	**<0.001**	0.970	0.953–0.996
GAD-7 total	**<0.001**	1.515	1.408–1.630
Fatigue				**<0.001**	1.338	1.219–1.469

Bolded values: <0.05; CI: confidence interval; OR: odds ratio.

Univariable analyses revealed that residents with depressive symptoms were more likely to be concerned about COVID-19 pandemic (*p* = 0.019), more worried about having COVID-19 infection (*p* < 0.001), had more economic loss during the COVID-19 wave (*p* < 0.001), had a history of psychiatric disorders (*p* < 0.001), and had suicidality during the COVID-19 wave (*p* < 0.001). Compared with those without depression, those with depression were less likely to have a higher monthly income (≥MOP30,000) (*p* < 0.001), and physical exercise 30 min or above every day during the pandemic (*p* = 0.001). Furthermore, the residents with depression were more likely to report a higher total score of GAD-7 (*p* < 0.001), fatigue (*p* < 0.001), and a lower QoL (*p* < 0.001). After controlling for variables with significant group differences in univariate analyses, residents with depression still had lower QOL (*F*_(1, 1,008)_ = 5.538, *p* = 0.019) compared to those without depression. Binary logistic regression analysis revealed that participants with depression were more likely to be younger (OR = 0.97; *p* < 0.001) and report more severe anxiety symptoms (OR = 1.515; *p* < 0.001) and fatigue (OR = 1.338; *p* < 0.001) and more severe economic loss (OR = 1.933; *p* = 0.026) during the wave (Table 2).

### 3.3. Network structure of depressive symptoms

**Figure 1:**
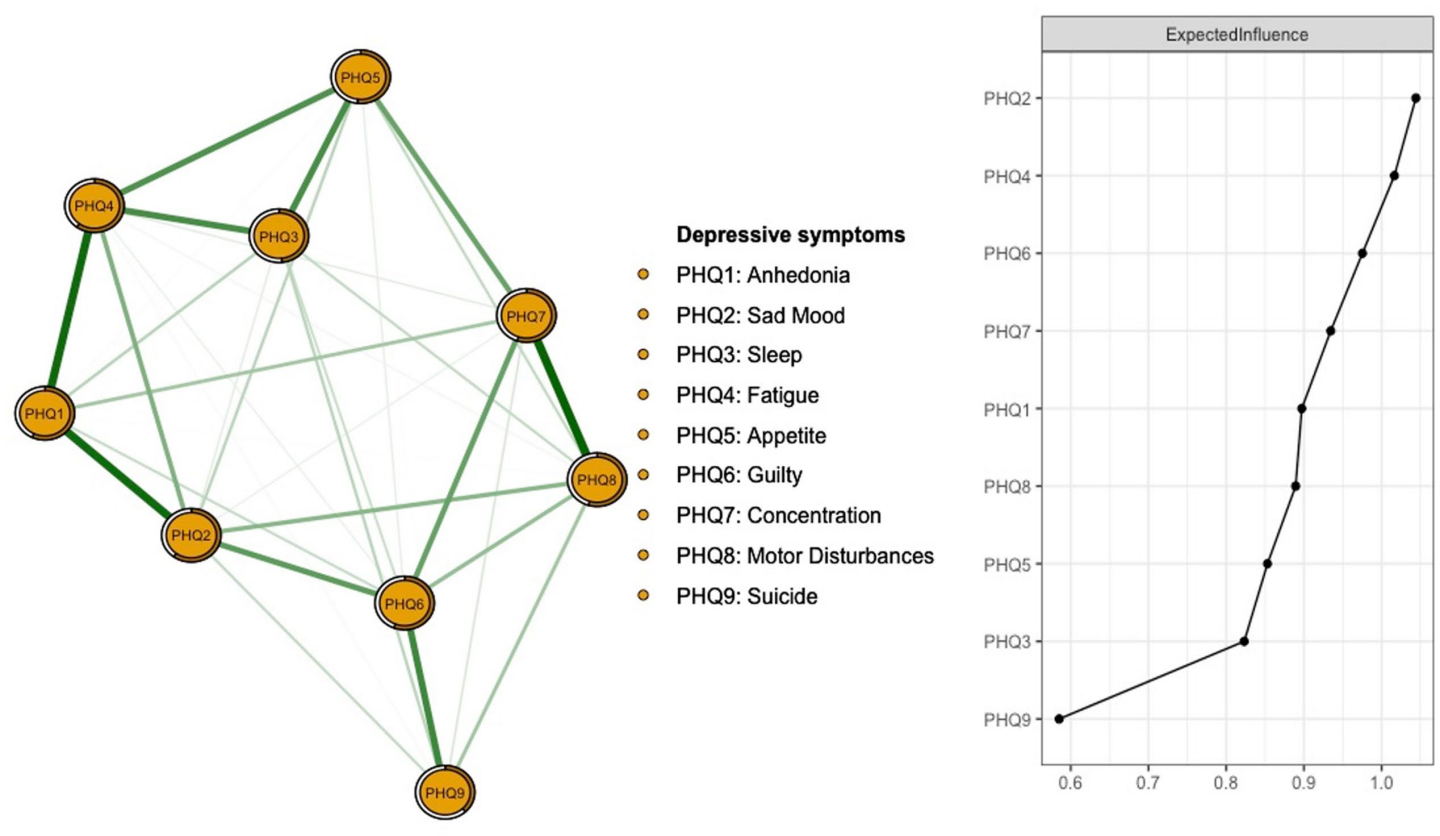
Network structure of depressive symptoms among Macau residents during the 618 COVID-19 wave. Number of nodes: 9; Number of non-zero edges:35/36; Mean weight: 0.111.

**Figure 2:**
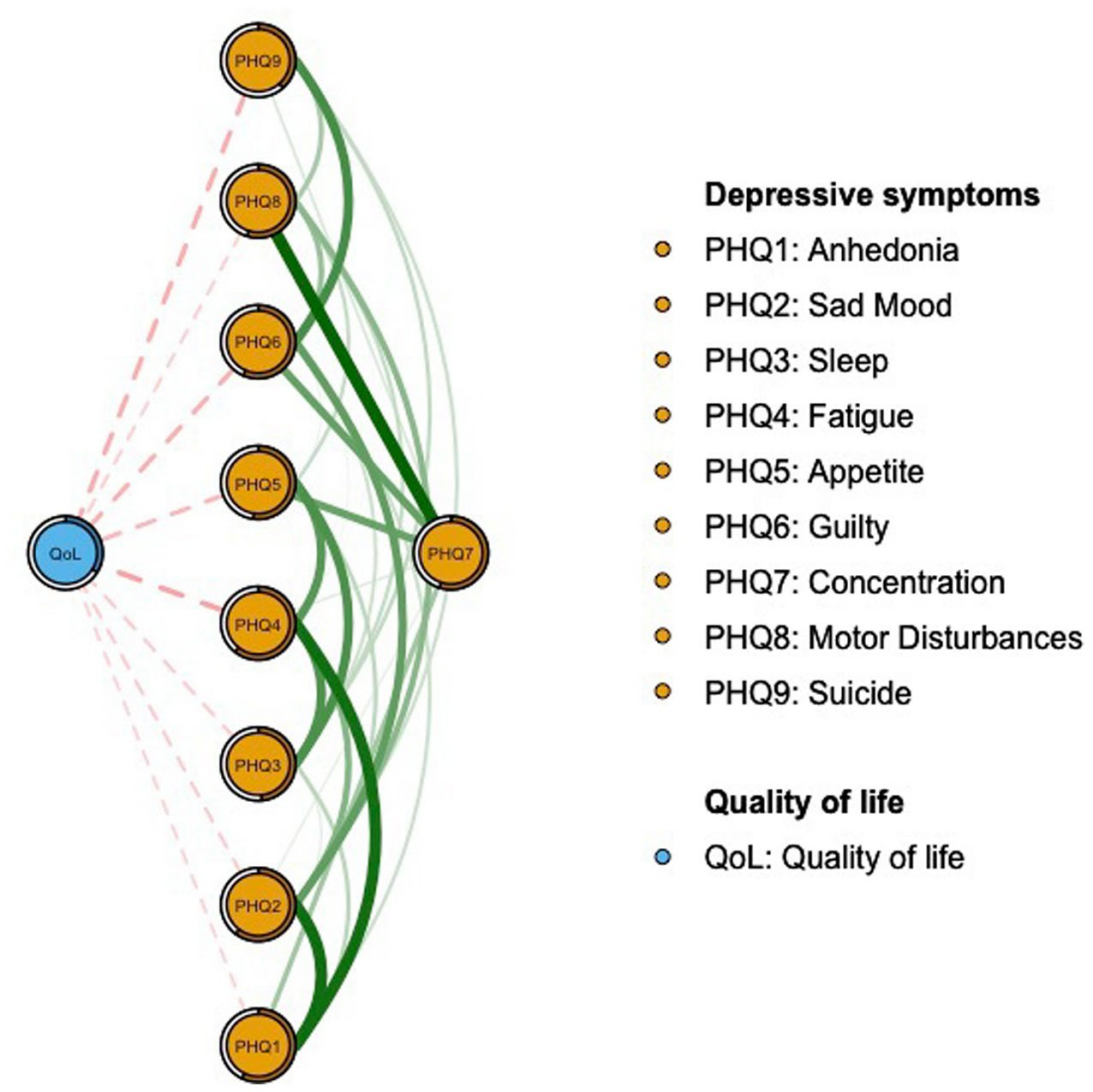
Flow network of quality of life and depressive symptoms.

The network structure of depressive symptoms as measured by PHQ items is shown in Figure 1. PHQ2 (“Sad Mood”; EI: 1.044), PHQ4 (“Fatigue,” EI: 1.016), and PHQ6 (“Guilt”; EI: 0.975) were the top three nodes with the highest EI. The mean predictability was 0.535, indicating that on average 53.5% of the variance for each node could be explained by neighboring nodes in the model. Supplementary Table S1 provides the descriptive details and network centrality indices for each depressive symptom. Figure 2 presents the results of flow network model showing that PHQ4 (“Fatigue”; average edge weight = −0.1162), PHQ9 (“Suicide”; average edge weight = −0.1029), and PHQ6 (“Guilt”; average edge weight = −0.0981) had strong negative associations with QoL.

**Figure 3:**
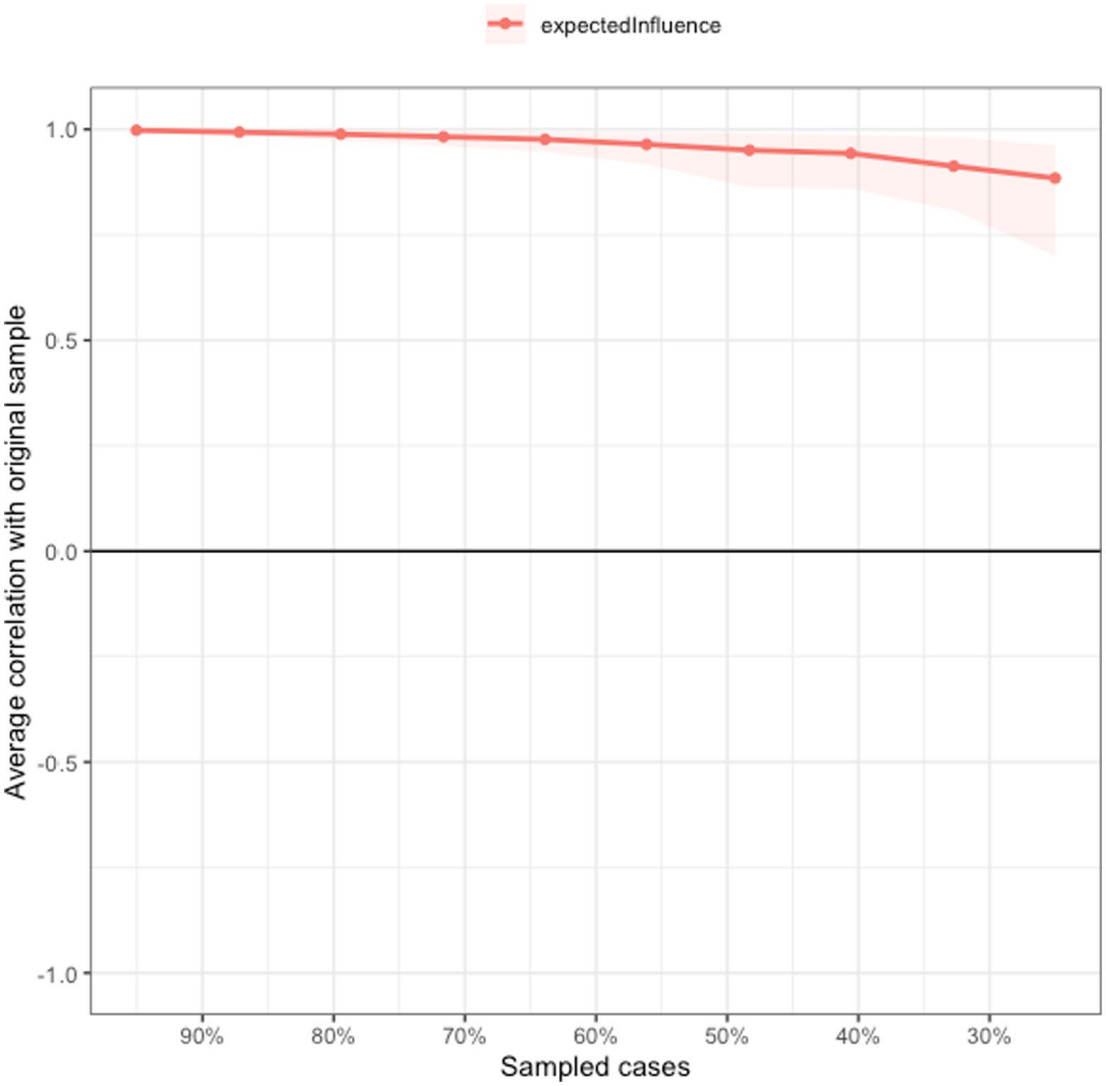
Network stability of depressive symptoms among Macau residents during the 618 COVID-19 wave.

Figure 3 shows the stability of the network. Based on the case-dropping bootstrap procedure, the CS-C of EI was 0.75, showing that the network model was very stable, which indicates that 75% of the sample could be dropped and the structure of the network would not significantly change (Figure 3). As shown in Supplementary Figure S1, the bootstrap 95% CI for estimating edge weights for the accuracy of the network indicated a limited range, and most of the edge weights were non-zero, indicating that most of the edges were stable and accurate. These comparisons were statistically significant based on a bootstrap difference test, showing the network model’s reliability (Supplementary Figure S2).

## 4. Discussion

This was the first study to explore the prevalence, related factors, and network structure of depression as well as its relationship with QoL of among Macau residents shortly after the 618 COVID-19 wave.

The prevalence of depressive symptoms (PHQ-9 total score ≥ 5) was 62.5% (95% CI = 60.0–65.0%) in Macau residents during the 618 COVID-19 wave, which was much higher than the corresponding figures (38.5%; 95% CI = 35.5%–41.5%) during the first wave of the COVID-19 infections in Macau (Bai et al., 2022b) and in most other areas such as the general population in mainland China (37.1%) (Ahmed et al., 2020), in Italy (32.8%) (Mazza et al., 2020) and in Hong Kong (48.8%) (Choi et al., 2020). The higher infection and death cases together with the more stringent public health measures during the 618 COVID-19 wave compared to the first wave of the COVID-19 infections in Macau could explain the higher prevalence of depression. Previous research showed that strict quarantine measures, social distancing, and isolation (Xiao, 2020; Ustun, 2021) could increase the risk of mental health problems including depression (Xiao, 2020).

More severe anxiety was associated with higher risk of depression in this study. During the 618 COVID-19 wave, mass media in Macau continuously reported the latest news about the wave, which caused stress for many people. High anxiety levels were associated with being constantly exposed to COVID-19 news (Moghanibashi-Mansourieh, 2020; WHO, 2020; Yu et al., 2023). A meta-analysis by Jacobson and Newman (2017) found that anxiety was closely associated with depression, and anxiety could often predict depression. We also found that participants who experienced a heavier economic loss during the COVID-19 wave were more likely to suffer from depressive symptoms, which is consistent with previous findings (Hertz-Palmor et al., 2020; Argabright et al., 2022). People who were economically disadvantaged were more likely to experience financial insecurity, unhealthy lifestyles, poor living conditions, and reduced social capital (Lund and Cois, 2018; Jin et al., 2020; Ding et al., 2022). All these factors might lead to a higher risk of depression. In addition, people with limited financial resources usually worry more about their financial insecurity, which could also lead to depression (Asebedo and Wilmarth, 2017).

Severe fatigue is another associated risk factor of depression. Compared with previous waves of the COVID-19 infections, the 618 COVID-19 wave lasted longer and had more stringent measures such as repeated nucleic acid testing, which could lead to subjective fatigue among residents. This might also be physiologically related due to increased stress hormones released in people in response to the rapidly growing 618 COVID-19 wave (Haktanir et al., 2022), which might result in fatigue over time. According to the psychological resource theory (Hobfoll, 2002) and the cognitive load theory (Sweller, 1988), people who experience fatigue with limited psychological resources may be less resilient to stress-related symptoms when subjected to ongoing negative mental or emotional states, which might, in turn, increase the risk of depression (Barsevick et al., 2006; Lee and Kim, 2006).

This study found a lower risk of depression (e.g., lower PHQ-9 scores) among older Macau residents, which is consistent with the findings of previous studies (Nwachukwu et al., 2020; García-Portilla et al., 2021). This might be attributed to the development of resilience or successful adaption to difficult or challenging life experiences, which is a personal characteristic that could be enhanced through practice (Southwick et al., 2014). Due to more exposure to stressful experiences compared to their young counterparts, older adults are usually more resilient (Silva Júnior et al., 2019; García-Portilla et al., 2021), particularly in terms of emotional regulation and problem-solving (Gooding et al., 2012). Hence, resilience in older adults are usually associated with positive outcomes, including adaptive coping, optimism, and a reduced risk of depression (Gooding et al., 2012; MacLeod et al., 2016).

“Sad mood” (PHQ2) was the most central symptom in the depression network model among Macau residents during the 618 COVID-19 wave, which is consistent with the previous findings in a German general population study (Hartung et al., 2019) and in a study of older adults in Hong Kong (Jin et al., 2022) during the COVID-19 pandemic. According to the Diagnostic and Statistical Manual of Mental Disorder-5 (DSM-5) (APA, 2013), “sad mood” is a core symptom of major depressive disorder (MDD), and persistent sadness is also a risk factor for depression (Wolff, 1999; Davidson and Henriques, 2000). Sadness was likely a normal and common reaction to a loss, disappointment, problem, or other difficult situations during the 618 COVID-19 wave.

Characterized by emotional tiredness, an inability to work efficiently, loss of motivation, difficulty falling asleep, helplessness, and resentment (Queen and Harding, 2020; Tanhan et al., 2020; Vindegaard and Benros, 2020), “Fatigue” (PHQ4) was also a central depressive symptom in the network model. This finding is consistent with the results of a study of college students during the late stage of the COVID-19 pandemic (Bai et al., 2021). Moreover, “guilt” (PHQ6) was another central symptom in the model among Macau residents, which is consistent with the findings among Hong Kong (Cheung et al., 2021) and Wuhan residents during the COVID-19 pandemic (Zhao et al., 2021). Guilt may manifest as helplessness, hopelessness, worthlessness, powerlessness, poor self-esteem, and self-doubt (Bademci et al., 2016; Gambin and Sharp, 2018), which may lead to negative outcomes such as a reduction in motivation and self-care (Luck and Luck-Sikorski, 2021) as well as depression. Further, guilt is not only closely related to grief, but also to suicidal thoughts (Jeon et al., 2014).

In the flow network model of QoL and depression, the top three symptoms that negatively correlated with QoL included “Fatigue” (PHQ4), “Suicide” (PHQ9), and “Guilt” (PHQ6). During the 618 COVID-19 wave, fatigue might be caused by lifestyle changes due to the lockdown measures and decreased outdoor physical activities (McIlvenny et al., 2000; Booth et al., 2012; Zhao et al., 2022b). As such, adequate physical exercise could improve physical performance and increase QOL (Mehnert et al., 2011; Jakobsen et al., 2017; Dauwan et al., 2021). Suicidality was another symptom which negatively correlated with QoL, which is expected since many people became vulnerable to mental health problems and suicidality during the pandemic (Gunnell et al., 2020; Luo et al., 2021). Loss of employment and financial stress during the pandemic are identified as risk factors for lowered QoL (Stuckler et al., 2009; Kokaliari, 2018; Norström et al., 2019). To improve QoL, the government needs to provide financial support (Gunnell et al., 2020), and maintain an active labor market program when necessary (Stuckler et al., 2009). Moreover, the symptom “guilt” (PHQ6) was also negatively associated with QOL in the network model. Untreated guilt caused by stressful events might be associated with severe mental health problems, which could lower QoL (Griffin et al., 2019; Cavalera, 2020). Previous studies found that sudden changes in the lifestyle of individuals due to social distancing and lockdown during COVID-19 pandemic increased feelings of guilt (Brooks et al., 2020; Cavalera, 2020; Sahoo et al., 2020), which could lower QoL (Tilghman-Osborne et al., 2012; Luck and Luck-Sikorski, 2021).

The strengths of this study included the relatively large sample size and the use of network analysis to explore the network structures of depression and the correlation between depression and QoL. However, this study has several limitations. First, this was a cross-sectional study; therefore, causal relationships between QoL and depressive symptoms could not be inferred. Second, the PHQ-9 is a self-report measure of depressive symptoms rather than a clinical interview assessment or diagnosis. The possibility of recall bias could not be excluded. Third, this study was conducted in Macau, hence the findings could not be generalized to other areas. Fourth, for logistical reasons, random sampling was not used in this study, which might bias the representativeness of the study sample to an uncertain extent.

In conclusion, depression was common among Macao residents during the 618 COVID-19 wave, particularly among older residents and those who had more severe anxiety, fatigue and economic loss. Due to the negative impact of depression on QoL, interventions targeting central symptoms (e.g., “Sad Mood”, “Fatigue”, “Guilt” and “Suicide”) identified in the network model should be developed and implemented for depressed Macau residents, such as the provision of public education, Internet counseling, and increased physical activity.

## Data availability statement

The datasets presented in this article are not readily available because the Institutional Review Board (IRB) of the University of Macau that approved the study prohibits the authors from disseminating the research dataset of clinical studies publicly. Requests to access the datasets should be directed to xyutly@gmail.com.

## Ethics statement

The studies involving human participants were reviewed and approved by Institutional Review Board (IRB) of the University of Macau. The patients/participants provided their written informed consent to participate in this study.

## Author contributions

LZ, YF, SS, and Y-TX study design. TS, PC, ML, K-IL, IC, J-XL, Y-YW, ZS, TC, and GU data collection, analysis, and interpretation. TS, PC, and Y-TX drafting of the manuscript. GU and CN critical revision of the manuscript. All authors contributed to the article and approved the submitted version.

## Funding

The study was supported by the National Science and Technology Major Project for investigational new drug (2018ZX09201-014), the Beijing Hospitals Authority Clinical Medicine Development of special funding support (XMLX202128), and the University of Macau (MYRG2019-00066-FHS and MYRG2022-00187-FHS).

## Conflict of interest

The authors declare that the research was conducted in the absence of any commercial or financial relationships that could be construed as a potential conflict of interest.

## Publisher’s note

All claims expressed in this article are solely those of the authors and do not necessarily represent those of their affiliated organizations, or those of the publisher, the editors and the reviewers. Any product that may be evaluated in this article, or claim that may be made by its manufacturer, is not guaranteed or endorsed by the publisher.
