# Supplementary material for: Depression and quality of life among Macau residents in the 2022 COVID-19 pandemic wave from the perspective of network analysis
Source: Front Psychol. 2023 Apr 24;14:1164232. doi: 10.3389/fpsyg.2023.1164232 (PMC10165090; doi:10.3389/fpsyg.2023.1164232)
Supplement: Supplementary file 1 [file Data_Sheet_1.docx]

**Supplementary materials**

Table S1. Descriptive information and network centrality indices of depressive symptoms

Figure S1. Bootstrapped confidence intervals of edge weights

Figure S2. Estimation of edge weight difference by bootstrapped difference test

Table S1. Descriptive information and network centrality indices of depressive symptoms

| Item | Item content | Mean (SD) | Prevalence ^a^ | Predictability | EI |
| --- | --- | --- | --- | --- | --- |
| PHQ1 | Anhedonia | 1.11 (0.928) | 72.7% | 0.572 | 0.897 |
| PHQ2 | Sad Mood | 0.93 (0.839) | 67.9% | 0.603 | 1.044 |
| PHQ3 | Sleep | 1.17 (1.003) | 71.4% | 0.485 | 0.812 |
| PHQ4 | Fatigue | 1.25 (0.993) | 79.5% | 0.603 | 1.016 |
| PHQ5 | Appetite | 0.85 (0.926) | 56.2% | 0.519 | 0.853 |
| PHQ6 | Guilt | 0.63 (0.915) | 39.7% | 0.561 | 0.975 |
| PHQ7 | Concentration | 0.68 (0.891) | 45.5% | 0.552 | 0.935 |
| PHQ8 | Motor disturbances | 0.49 (0.790) | 33.9% | 0.549 | 0.889 |
| PHQ9 | Suicide | 0.21 (0.594) | 14.4% | 0.370 | 0.585 |

Note:

^a^ The prevalence indicates the rate of the presence of each symptom with a score more than 0.

IQR: interquartile range; SD: standard deviation; EI: Expected influence.

Figure S1. Bootstrapped confidence intervals of edge weights


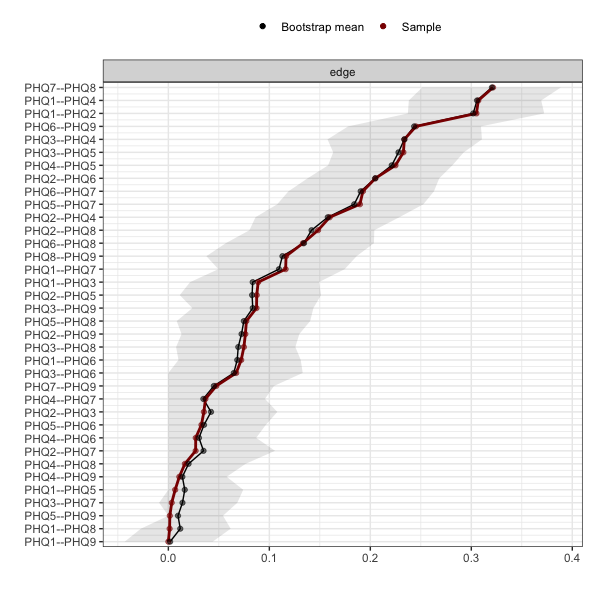


Notes: The black dots indicate the values of each edge weight, ordered from the highest to the lowest value. The gray area represents the 95% confidence intervals of edge weights, estimated with the non-parametric bootstrap procedure. (PHQ1: Anhedonia; PHQ2: Sad Mood; PHQ3: Sleep; PHQ4: Fatigue; PHQ5: Appetite; PHQ6: Guilt; PHQ7: Concentration; PHQ8: Motor disturbances; PHQ9: Suicide. )

Figure S2. Estimation of edge weight difference by bootstrapped difference test


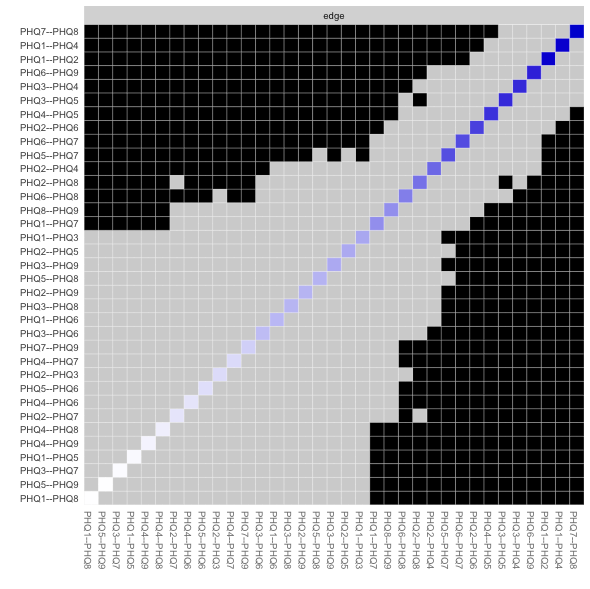


Notes: Gray boxes indicate edges that do not significantly differ from one-another. Black boxes represent edges with significant difference from one another (α = 0.05). Blue boxes in the edge-weight plot indicate positive correlations.
